# Ultrasound-Assisted Pressurized Fluid Extraction of Antioxidant and Anticancer Molecules from a Mangaba, Cambuí and Red Propolis Blend

**DOI:** 10.3390/molecules30193857

**Published:** 2025-09-23

**Authors:** Diego S. de Oliveira, Marília R. Oliveira, Glenda A. da Silva, Cristiane B. Corrêa, Ana Veruska C. da Silva, Jhonattas de C. Carregosa, Alberto Wisniewski, Maria Beatriz P. P. Oliveira, Claudio Dariva, Klebson S. Santos

**Affiliations:** 1 Center for Study on Colloidal Systems (NUESC), Institute of Research and Technology and (ITP), Tiradentes University (UNIT), Av. Murilo Dantas, 300, Aracaju 49032-490, SE, Brazil; mestrado_diegosantos@souunit.com.br (D.S.d.O.); marilia_santos@itp.org.br (M.R.O.); jhonattas.carregosa@live.com (J.d.C.C.); claudio_dariva@itp.org.br (C.D.); 2 Department of Morphology, Federal University of Sergipe (UFS), São Cristóvão 49100-000, SE, Brazil; glendaamaral@academico.ufs.br (G.A.d.S.); crisbani@gmail.com (C.B.C.); 3 Empresa Brasileira de Pesquisa Agropecuária (EMBRAPA), Av. Beira-Mar, 3.250, Aracaju 49025-040, SE, Brazil; ana.veruska@embrapa.br; 4 Petroleum and Energy from Biomass Research Group (PEB), Department of Chemistry, Federal University of Sergipe (UFS), São Cristóvão 49100-000, SE, Brazil; albertowj@academico.ufs.br; 5 REQUIMTE/LAQV, Department of Chemical Sciences, Faculty of Pharmacy, University of Porto, R. Jorge de Viterbo Ferreira, 228, 4050-313 Porto, Portugal

**Keywords:** bioactive compounds, *Myrciaria floribunda*, *Hancornia speciosa*, pressurized liquid extraction, sonication, green solvents, lung cancer

## Abstract

This study explored the antioxidant and anticancer potential of extracts obtained from the mangaba, cambuí, and red propolis blend. The extracts were obtained using ultrasound-assisted pressurized fluid extraction (UAPFE) at 50 bar, 60 °C, and a flow rate of 2 mL/min. Both sequential extraction with solvents of increasing polarity (propane followed by ethanol/water) and one-step extraction were employed for 30 min. Extracts were characterized by ultra-high-resolution mass spectrometry, total phenolic content, antioxidant activity (via DPPH and FRAP assays), and cytotoxicity using the sulforhodamine B colorimetric method. Among the tested conditions, the sequential extraction with ethanol/water (UAPFE-SE) yielded 16.2 ± 3.0% (overall extraction yield), with high phenolic content (24.1 ± 0.4 µg/mg). Mass spectrometry revealed the presence of antiproliferative phenolics. The UAPFE-SE extract demonstrated moderate antioxidant activity, with FRAP values of 394.0 ± 6.0 µg Fe^2+^/mg and DPPH scavenging capacity of 28.5 ± 0.3 µg Trolox equivalents/mg. Additionally, it exhibited cytotoxic inhibition of 82.3 ± 1.7% against lung carcinoma cells at a concentration of 100 μg/mL. The results suggest that the antioxidant properties and cytotoxic effect against lung cancer cells in vitro warrant further investigation to assess therapeutic potential.

## 1. Introduction

The growing interest in health beneficial products has boosted the value of bioactive compounds derived from natural sources. This trend stems from the vast chemical diversity found in plant matrices, which display a wide range of biological activities, including antioxidant, antibacterial, antiviral, anti-inflammatory, and antitumor effects [1].

Medicinal plants from Brazilian flora are valuable sources for therapies with low toxicity and high selectivity against cancer cells. Red propolis, a unique resinous substance harvested by bees in northeastern Brazilian mangroves, contains bioactive compounds such as flavonoids and phenolic acids with anti-inflammatory, antioxidant, antimicrobial, and anticancer properties [2,3,4]. Mangaba (*Hancornia speciosa* Gomes), a South American tropical plant, produces fruits rich in ascorbic acid and flavonoids, demonstrating strong antioxidant activity and potential anticancer effects [5,6].

Cambuí (*Myrciaria floribunda* H.West ex Willd. O.Berg), a native species of South America, produces fruits with high concentrations of phenolic compounds, which are known for their potent biological activities [7,8]. Preliminary studies suggest that this phytochemical profile may confer promising pharmacological properties, including antioxidant and anti-inflammatory effects [9].

The practical application of bioactive molecules depends heavily on selecting the right solvents and extraction techniques that preserve chemical integrity and maximize yield [1]. Hence, solvents such as propane, ethanol, water, and carbon dioxide are effective solvents for extracting bioactive compounds, and ethanol/water mixtures can enhance the affinity and solubility of polyphenols [10].

Among the various extraction methods, such as maceration, Soxhlet extraction, and hydrodistillation [1,11], as well as more advanced techniques like microwave-assisted extraction, supercritical fluid extraction [1,12], ultrasound-assisted extraction (UAE), and pressurized liquid extraction (PLE) stand out for their high efficiency. When paired with selective solvents, these methods significantly enhance the recovery of bioactive compounds from complex plant matrices [1,12]. Notably, combining PLE with UAE can further optimize extraction performance and improve solvent selectivity [13,14].

This study explores the combined extract from red propolis, mangaba, and cambuí. These three natural resources are individually known for their antioxidant, anti-inflammatory, antimicrobial, and anticancer properties. Unlike previous studies that focus on each component separately, this study addresses a critical gap by evaluating their combined effects against cancer cell lines, particularly lung cancer. Lung cancer is a major cause of global morbidity and mortality, and crucially lacks more effective treatments [7,9].

The approach of the present study is based on the pharmacological principle that complex diseases like cancer involve multiple dysregulated pathways, making multi-target therapies more effective than single-target ones. Natural products, owing to their remarkable chemical diversity, often exhibit multi-targeted biological activities. Among these, potent antioxidant effects play a crucial role in mitigating oxidative stress, a key contributor to cancer progression [15].

By combining these three vegetable matrices, this study represents a shift from the conventional one drug/one target model toward intelligent, multi-component natural formulations for treating complex diseases. Moreover, the systematic application of sequential ultrasound-assisted pressurized fluid extraction (UAPFE) to a complex blend for obtaining antioxidant and antiproliferative compounds constitutes a methodological advancement. Thus, the present study aims to obtain antioxidant and antiproliferative molecules with action against lung cancer cells from a vegetal blend and contribute to the development of safer, more effective, and sustainable therapeutic alternatives to combat cancer.

## 2. Results

### 2.1. Overall Extraction Yield (OEY)

**Figure 1:**
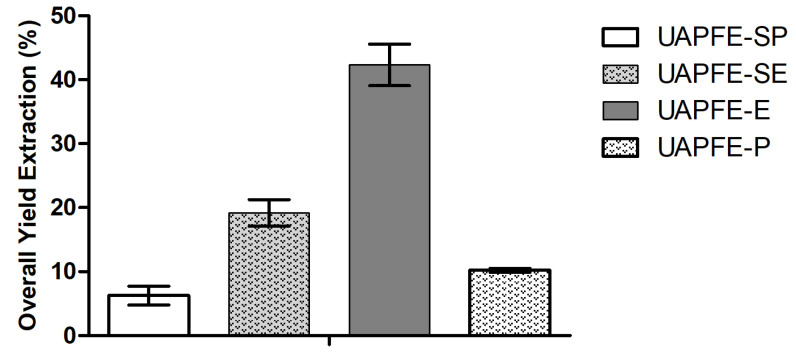
OEY obtained from the UAPFE process. Extractions were performed under 50 bar pressure, at 60 °C, and a flow rate of 2 mL/min. All results are expressed as mean ± standard deviation from three independent experiments.

Figure 1 presents the OEY obtained through ultrasound-assisted pressurized fluid extraction of the red propolis, mangaba, and cambuí blends using propane and ethanol/water (80:20, *v*/*v*) as solvents. The extractions were carried out in two modes: the first one was a 30 min one step extraction conducted with single solvents: ethanol (UAPFE-E) and propane (UAPFE-P). In the sequential process, the 30 min of extraction were divided for the two solvents: propane was used in the first 15 min (UAPFE-SP), followed by ethanol in an additional 15 min of extraction (UAPFE-SE). The ethanol/water solvent resulted in significantly higher OEY compared to propane, as observed in both UAPFE and sequential UAPFE methods.

The OEY obtained with UAPFE-E (42.3 ± 3.2%) was significantly higher than that achieved with UAPFE-P (10.2 ± 0.31%). This finding indicates that the ethanol/water solvent system is markedly more effective than propane under the evaluated conditions. This superiority was consistently observed in both single-step extractions (30 min per solvent) and sequential extractions. Sequential extractions yielded 16.2 ± 3.0% for UAPFE-SE and 6.3 ± 1.5% for UAPFE-SP, both performed for 15 min per solvent, totaling 30 min. A comparison between the ethanol/water-based sequential extraction (UAPFE-SE: 16.2 ± 3.0%) and its one-step counterpart (UAPFE-E: 42.3 ± 3.2%) revealed that the single-step method produced a significantly higher yield. This result suggests that a continuous 30 min extraction with the ethanol/water blend may facilitate more efficient solubilization and recovery of overall compounds than a sequential extraction using two solvents with distinct polarities.

### 2.2. Molecular Characterization of Extracts Obtained via Sequential and One-Step UAPFE Using HESI(±)-FT Orbitrap Mass Spectrometry

The blend extracts obtained through combinations of UAPFE processes showed distinct molecular profiles. When considering ions with a signal-to-noise ratio greater than 3 (S/N > 3), the ethanolic extracts revealed 189 and 149 molecular ions for the UAPFE−E and UAPFE−SE processes, respectively. Similarly, the propane extracts presented 165 and 158 molecular ions for the UAPFE−P and UAPFE−SP processes.

This reduction in the number of detected molecules following the sequential extraction process suggests a decrease in extract complexity, particularly for neutral and acidic compounds (negative ionization mode). In contrast, for neutral and basic compounds analyzed in positive ionization mode, the sequential process led to an increase in molecular complexity. Specifically, the ethanolic extracts showed 331 and 366 molecular ions for UAPFE−E and UAPFE−SE, respectively, while the propane extracts yielded 341 and 374 molecular ions for UAPFE−P and UAPFE−SP.

**Figure 2:**
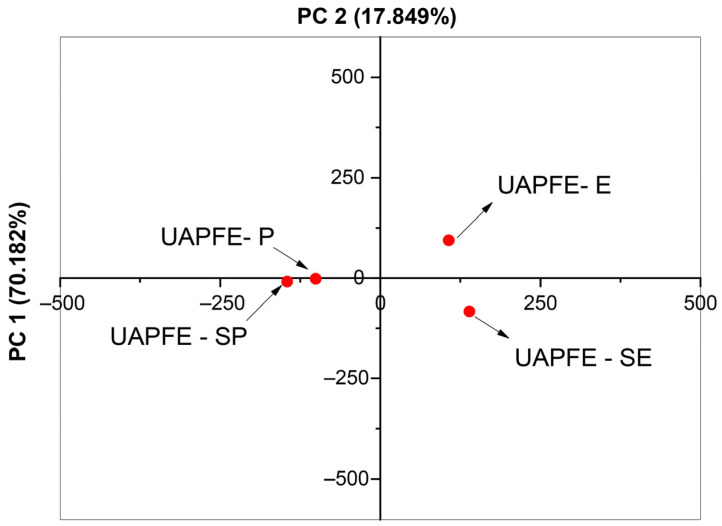
Principal Component Analysis (PCA) score plots illustrating the clustering and separation of vegetable blend extracts using different solvents (ethanol and propane) and processes (sequential and one-step UAPFE). Molecular profiles were obtained via HESI(±)-FT Orbitrap Mass Spectrometry.

As shown in Figure 2, the PCA score plot (PC1 ~70%) revealed distinct clustering of samples, indicating differentiation based on chemical composition. Samples extracted with the same solvent were grouped together, reflecting compositional similarity. Furthermore, the second principal component (PC2 ~18%) highlighted the impact of the sequential process, particularly on ethanolic extracts. This is evidenced by the greater Euclidean distance between UAPFE−E and UAPFE−SE compared to the distance between UAPFE−P and UAPFE−SP, suggesting a more pronounced chemical shift induced by the sequential ethanol extractions.

To evaluate the PC1 loadings, a subsequent statistical treatment was applied to the dataset. A Gaussian distribution curve was constructed for the 1.911 loadings, and only those falling outside the 95% confidence interval were considered statistically significant. From this analysis, 19 loadings were identified as significant, representing the influence of solvent type and extraction process on the molecular composition of the extracts. These PC1 loadings correspond to neutral molecules, as determined by molecular ions detected via HESI(±)-FT Orbitrap MS. Notably, these ions were exclusively observed in the negative ionization mode. The compounds deemed representative are listed in Table 1, along with their relative percentage area (Area %), indicating their abundance and contribution to extract composition. Based on literature reports of compounds previously identified in the isolated components of the blend, several molecules listed in Table 2 were tentatively identified and classified. Their respective blend sources, known biological activities, and potential applications are summarized.

### 2.3. Total Phenolic Compounds (TPC)

**Figure 3:**
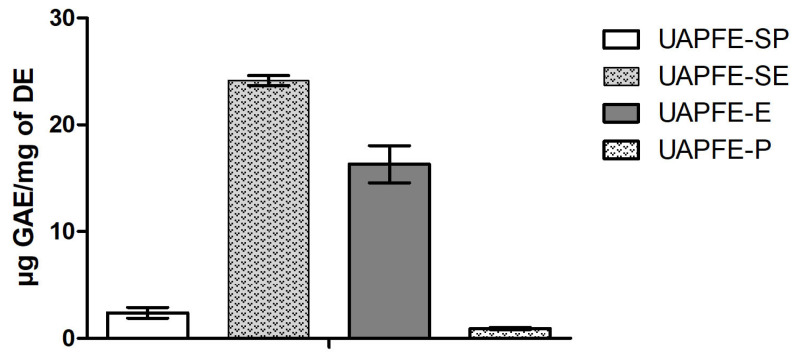
Total Phenolic Content of UAPFE extracts, expressed as µg gallic acid equivalents (GAE)/mg of dry extract (DE). Values are shown as mean ± standard deviation.

The TPC varied significantly depending on the solvent system and the extraction process (sequential vs. one-step UAPFE), ranging from 0.9 to 24.1 μg gallic acid equivalents (GAE) per mg of DE (Dry extract). The highest concentrations were observed in extracts obtained using ethanol/water (80:20, *v*/*v*), specifically in UAPFE-E and UAPFE-SE, while the lowest concentrations were found in UAPFE-P and UAPFE-SP (Figure 3). These results indicate that the ethanol/water mixture was significantly more effective than propane (UAPFE-P and UAPFE-SP) in extracting TPC from the vegetable blend.

Based on a statistical analysis of the results between the obtained extracts, it was noted that there was a significant amount of phenolic compounds in the sequential extraction with ethanol/water (UAPFE-SE: 24.1 ± 0.4 µg GAE/mg of DE) when compared to extracts obtained with the same solvent but in a one-step (UAPFE-E: 16.3 ± 2.0 µg GAE/mg of DE). Similarly, in extractions performed with propane, the sequential mode (UAPFE-SP: 2.4 ± 0.3 µg GAE/mg of DE) obtained a significantly higher concentration of total phenols compared to extraction in a one-step (UAPFE-P: 0.9 ± 0.1 µg GAE/mg of DE). It is hypothesized that the 30 min one-step extractions led to a dilution of the phenolic compound concentration. This outcome may be attributed to the co-extraction of other compounds during the extended time, as the one-step extraction (30 min) was twice as long as the initial propane and ethanol/water steps in the sequential extraction (15 min). This extended duration subsequently lowered the concentration of TPC from the cambuí, mangaba, and red propolis blend. The results in Figure 3 also suggest that the use of propane before the ethanol/water (80:20, *v*/*v*) extraction acts as a preliminary cleaning step. This initial extraction removes non-phenolic compounds from the matrix, thereby enhancing the interaction and recovery of phenolic compounds during the subsequent hydroethanolic extraction.

### 2.4. Assessment of DPPH Radical Scavenging

**Figure 4:**
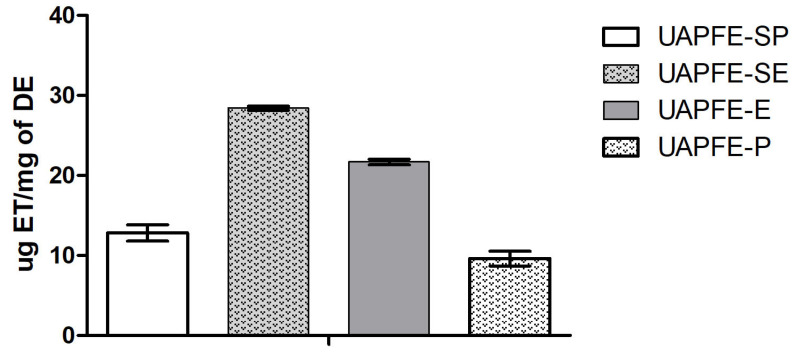
Antioxidant activity of vegetable blend extracts obtained via sequential and one-step UAPFE, as determined by the DPPH radical scavenging assay. Data are presented as the mean percentage of DPPH inhibition ± standard deviation.

The antioxidant activity of the UAPFE extracts was determined by the DPPH method, which assesses their radical scavenging capacity. The results, presented in Figure 4, are reported as Trolox equivalent antioxidant capacity (TEAC). Given the absence of literature data for direct comparison with this unique plant blend, the UAPFE extracts antioxidant activity was benchmarked against a commercial Trolox standard.

Based on the results obtained, the UAPFE-SE extract exhibited significantly greater antioxidant activity (28.5 ± 0.3 μg ET/mg of DE) compared to the single-step extraction using the same solvent (UAPFE-E: 22.0 ± 0.2 μg ET/mg of DE). A similar trend was observed for the propane extractions, where the sequential method (UAPFE-SP: 12.0 ± 1.0 μg ET/mg of DE) showed considerably higher antioxidant activity than the single-step extraction (UAPFE-P: 9.0 ± 0.4 μg ET/mg of DE).

### 2.5. Determination of FRAP

**Figure 5:**
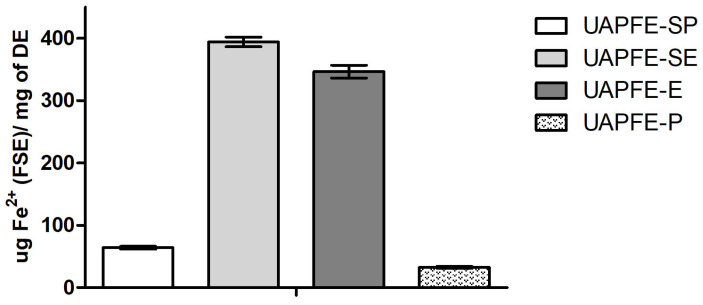
Antioxidant activity by the FRAP method of vegetable blend extracts obtained from sequential and one-step UAPFE. Values are expressed as mean ± standard deviation.

The antioxidant potential of the vegetable blend extracts obtained by UAPFE was evaluated through FRAP analysis (Figure 5). Based on the results in Figure 5, the sequential UAPFE-SE extract showed the highest FRAP values (394.0 ± 6.0 µg Fe^2+^/mg of DE) compared to the direct UAPFE-E extraction (346.5 ± 7.0 µg Fe^2+^/mg of DE) using the same solvent. Similarly, with propane, the sequential UAPFE-SP extract (64.0 ± 4.1 µg Fe^2+^/mg of DE) also yielded significantly higher values than the direct UAPFE-P extract (30.2 ± 2.1 µg Fe^2+^/mg of DE).

The results presented in Figure 5 suggest a direct correlation between the concentration of TPC in the sequential UAPFE vegetable blend extracts and their antioxidant activity. This observation supports the hypothesis that direct extraction methods (UAPFE-E and UAPFE-P) are less selective, potentially co-extracting non-phenolic compounds that do not contribute to the observed activity (Figure 4 and Figure 5). Therefore, these findings confirm that the extraction process significantly impacts the antioxidant properties of the final product.

### 2.6. Evaluation of the Cytotoxicity of the Vegetable Blend Extracts

**Figure 6:**
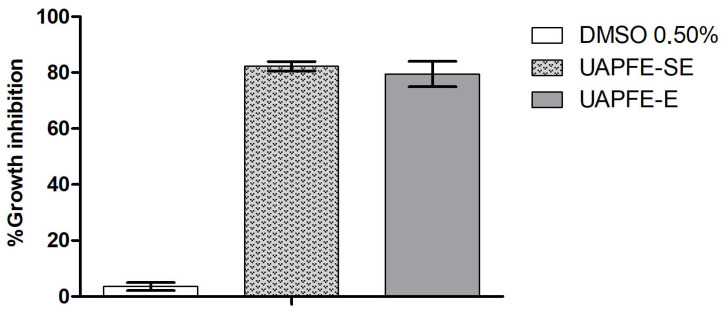
Cytotoxic Activity of UAPFE Ethanolic Extracts (100 µg/mL) Against A549 Cells. Values are expressed as mean ± standard deviation of the %cell growth inhibition.

The vegetable blend extracts obtained with propane (UAPFE-P and UAPFE-SP) were not tested for cytotoxic activity against A549 human lung carcinoma cells. This decision was based on their significantly lower content of phenolic compounds (Figure 3) and antioxidant activity (Figure 4 and Figure 5) compared to the ethanolic extracts (UAPFE-E and UAPFE-SE). Based on the data presented in Figure 6, no statistically significant differences in cytotoxic activity were observed between the UAPFE-E (79.5 ± 4.5%) and UAPFE-SE (82.3 ± 1.7%) extracts, both obtained using a hydroethanolic solvent system (ethanol/water, 80:20). These findings suggest that the variation in extraction methodology UAPFE-E (one-step extraction) versus UAPFE-SE (sequential extraction) did not result in extracts with a statistically superior or inferior inhibitory effect on A549 human lung carcinoma cells under the tested conditions.

The results shown in Figure 6 indicate that both UAPFE-E and UAPFE-SE approaches are comparably effective in terms of cytotoxic potential. However, it is noteworthy that the UAPFE extracts exhibited a pronounced inhibitory effect on A549 human lung carcinoma cells at a concentration of 100 μg/mL.

## 3. Discussion

Obtaining bioactive compounds from plant matrices is a complex process. Key factors, such as the extraction method, time, temperature, and solvent type, directly influence both the overall yield and the selectivity of the extracted compounds [37]. The choice of solvent, in particular, is critical, as it dictates the efficiency of the process based on the polarity of the target compounds. Low-polarity compounds have a greater affinity for nonpolar solvents, whereas polar compounds, like the phenolics found in this study’s plant blend, are more efficiently extracted by polar solvents due to intermolecular interactions [38].

The application of an ethanol/water hydroalcoholic solvent system is consistent with the polarity characteristics of the target phenolic compounds [37]. Phenolic acids, flavonoids, and terpenes, frequently encountered in plant-derived matrices, exhibit enhanced solubility in hydroalcoholic mixtures compared to pure ethanol [39]. In the sequential extraction process adopted in this study, comprising an initial nonpolar propane phase followed by a polar ethanol/water step, increased selectivity for bioactive constituents was achieved (Figure 2 and Figure 3). The propane phase was used to remove nonpolar compounds like waxes, lipids, and apolar bioactive compounds, while the subsequent ethanol/water phase enriched the extract with molecules of pharmacological relevance. This two-step purification strategy contributed to both compositional refinement and improved functional quality of the final extracts [40,41,42].

In contrast, one-step extractions utilizing either propane or ethanol/water tend to maximize overall yield by solubilizing a broader spectrum of compounds (Figure 2 and Table 2). However, this approach may also co-extract non-target constituents that could compromise the desired biological activity [40,41,42]. This interpretation is supported by the propane-only extractions, which exhibited no statistically significant differences in overall extraction yield (Figure 1), indicating that the mass increase observed in the sequential process is primarily attributable to the hydroalcoholic phase [43,44].

Pressure is another key variable. High pressures in pressurized liquid extraction (PLE), especially above 100 bar, can compact the plant matrix, hinder solvent diffusion, and reduce extraction efficiency [45,46]. In the case of ultrasound-assisted PLE (PLEUS), pressures above 100 bar can negatively affect bubble dynamics, minimizing the beneficial effects of the ultrasound [1,13]. For example, Merma et al. (2022) [46] reported higher yields at 40–80 bar compared to 100 bar. Similarly, Santos, Veggi & Meireles (2012) [47] found optimal conditions for extracting anthocyanins and phenolic compounds at 50 bar, reinforcing that moderate pressures tend to favor both the yield and the selectivity of target compounds.

Because no prior research has specifically examined this particular plant blend, our findings were contextualized by comparing them to data from its individual components. Reis et al. (2020) [48] reported a very high overall yield (63%) from red propolis using supercritical CO_2_ with ethanol. This result, while impressive in terms of quantity, likely includes a significant proportion of undesirable compounds like waxes and lipids, which can inflate the total mass without contributing to the desired bioactive profile [44,49]. Our approach, while potentially yielding a smaller mass, focuses on a higher concentration of the molecules of interest.

From an analytical standpoint, plant extracts are chemically complex, containing a vast number of molecules [50]. While chromatographic methods are useful for targeted quantification, their time-consuming nature and cost make them less ideal for comprehensive analysis. Ultra-high-resolution mass spectrometry with direct infusion offers a more rapid and comprehensive approach for differentiating molecular profiles [51,52]. Multivariate statistical analysis, specifically Principal Component Analysis (PCA), played a pivotal role in this study by enabling the evaluation of solvent and process-dependent variations in extract composition. As illustrated in Figure 2, the PCA score plot revealed distinct clustering patterns, reflecting the influence of extraction parameters on the chemical profiles of the samples. This analysis effectively explained the largest variance in our data, providing a robust model for understanding the chemical diversity of the extracts [53].

The blended extracts analyzed in this study contained multiple classes of bioactive compounds, including flavonoids, chalcones, benzophenones, and sugars (Table 2). While the majority of these constituents originated from red propolis, relevant bioactive molecules were also identified in the other botanical matrices. For example, formononetin, a major isoflavone in red propolis, has demonstrated anticancer activity [23], whereas quinic acid, predominantly found in cambuí, exhibits antioxidant, antidiabetic, and anticancer properties [9,35]. The ultrasound-assisted pressurized fluid sequential extraction (UAPFE-SE) process employed in this study proved particularly effective in obtaining these compounds, indicating high selectivity for flavonoids (Table 2), which are strong candidates for the observed antioxidant and cytotoxic effects.

The comparative performance of the tested extraction methods reveals a fundamental trade-off between extract yield and phenolic enrichment. Sequential extraction processes, such as UAPFE-SE (sequential ethanol/water extraction) and UAPFE-SP (sequential propane extraction), promote selectivity for phenolic compounds (Figure 3). This is attributed to the preliminary removal of nonpolar constituents, which minimizes interference and enhances the recovery of target molecules from the red propolis, mangaba, and cambuí blends.

In contrast, one-step extractions, especially those using polar solvents with extended contact times, facilitate the broader solubilization of various matrix components (Figure 1). This approach typically results in a higher overall yield but may lead to lower concentrations of specific bioactive compounds (Figure 3). This dynamic highlights the critical need to align the chosen extraction strategy with the intended application.

For formulations designed to maximize biological potency, such as for antioxidant or cytotoxic activities, the selective enrichment of phenolics is a primary objective. In this regard, the sequential methods demonstrated a superior phenolic profile (Figure 3) and are therefore recommended for obtaining extracts with enhanced bioactivity. Conversely, if the objective is to maximize extract mass for industrial or nutritional applications, single-step methods may offer greater process efficiency. Ultimately, the decision between a sequential or single-step extraction should be guided by a careful balance between compositional precision and operational yield, a choice that is ultimately dictated by the intended therapeutic or functional application of the extract.

The antioxidant potential of the extracts, assessed via DPPH (Figure 4) and FRAP (Figure 5) assays, was closely associated with the presence of phenolic compounds. The results confirmed that sequential extraction enhances both selectivity and extract quality, whereas one-step extractions may co-extract non-phenolic constituents such as terpenes and alkaloids, potentially diluting the targeted biological activity [54,55]. Variability in antioxidant values reported across studies may be attributed to differences in analytical methodologies, seasonal variation, and the intrinsic chemical composition of the plant materials [56].

The relevant antioxidant and cytotoxic activities of the blend extracts against lung cancer cells are promising. The antioxidant activity is linked to compounds like pi-nobanksin, formononetin, quinic acid, and chalcones, with phenolic compounds being the most prevalent. These compounds act as hydrogen donors, reducing oxidative damage and inflammation [57]. This is a crucial point, as bioactive interactions between natural products and existing drugs are important for developing more effective therapies [58].

The antiproliferative activity of the blended extract against A549 human lung carcinoma cells observed in this investigation (Figure 6) is consistent with existing literature on the individual components. The strategic selection of a blended extract incorporating red propolis, cambuí, and mangaba is strongly supported by a robust body of scientific literature that extensively documents the individual bioactivities of each constituent (Table 2). These natural products are widely recognized for their potent cytotoxic and antioxidant properties, characteristics that are particularly pertinent to the investigation of cancer cell inhibition and the modulation of oxidative stress.

Red propolis has consistently demonstrated significant cytotoxic potential. Mendonça et al. (2015) [26], for instance, reported near-complete inhibition of the SF-295 (100%), OVCAR-8 (93.54%), and HCT-116 (98.12%) cell lines at a concentration of 50 µg/mL. More recently, Ghazy and Hanafy (2024) [59] provided further evidence by showing that propolis-loaded nanoparticles exerted pronounced antiproliferative effects on MCF-7 and A549 cells, thereby reinforcing their therapeutic relevance. Complementing this, extracts derived from cambuí leaves have also exhibited promising antiproliferative activity. Tietbohl et al. (2017) [60] documented total growth inhibition (TGI) across seven human cancer cell lines, with ethyl acetate extracts yielding TGI values ranging from 69.70 to 172.10 µg/mL, highlighting the species’ capacity as a source of valuable bioactive metabolites.

The inclusion of mangaba significantly contributes to the therapeutic profile of the blend. Its ethanolic leaf extract has been observed to induce apoptosis in Kasumi-1 leukemia cells, with late apoptosis rates escalating from 21.6 to 78.7% at concentrations of 100 and 200 µg/mL, respectively, Santos et al. (2016) [57]. Moreover, PEG microspheres loaded with mangaba fruit extract displayed antitumoral activity against co-cultured MCF-7 breast cancer cells, maintaining cell viability above 90% even at minimal concentrations of 50 and 100 ng/mL [61].

Mass spectrometry analysis of the blended extract identified several bioactive compounds with well-established anticancer and antioxidant properties, including formononetin, thevetiaflavone, and naringenin (Table 2). These findings are consistent with previous studies on the chemical composition of red propolis and the efficiency of pressurized fluid extraction systems in obtaining such compounds [26,62,63].

Given that a substantial proportion of currently approved anticancer agents are derived from natural sources [64,65,66,67], the identification of these compounds reinforces the therapeutic relevance of the blend. The data presented herein support the hypothesis that combining individual vegetable matrices not only preserves their intrinsic bioactivities but may also enhance them through interactions of bioactive compounds. This concept is in line with emerging strategies in phytochemical research, which increasingly focus on the cumulative and interactive effects of complex plant matrices.

Accordingly, the use of a vegetal blend represents a practical and strategically advantageous alternative to the isolated evaluation of single extracts. This approach addresses key limitations associated with single-compound analysis, such as scalability and biological variability, while offering a more integrative perspective on the therapeutic potential of natural products.

## 4. Materials and Methods

### 4.1. Chemical Products

Milli-Q water and various chemical reagents were sourced from different suppliers. Gallic acid, sodium acetate, Trolox, Folin–Ciocalteu phenol reagent, DPPH (2,2-diphenyl-1-picrylhydrazyl), absolute ethanol, TPTZ (2,4,6-tripyridyl-s-triazine) solution, and ferrous sulfate heptahydrate were all obtained from Sigma–Aldrich (St. Louis, MI, USA). TRIS base was purchased from Inlab Confiança (São Luís, Brazil), and sodium carbonate from Merck (Darmstadt, Germany). Propane (minimum 99.5% purity in the liquid phase) was purchased from White Martins SA (São Paulo, Brazil).

### 4.2. Sample Collection and Processing

Samples of mangaba (*Hancornia speciosa* Gomes), cambuí (*Myrciaria floribunda* H. West ex Willd. O. Berg) fruits, and red propolis were used in this study. Ripe mangaba fruits were collected from the Active Germplasm Bank of Embrapa Tabuleiros Costeiros, located in Itaporanga D’Ajuda, Sergipe, Brazil (11°06′40″ S; 37°11′15″ W). Ripe cambuí fruits were harvested from a native population within the Caju Private Natural Heritage Reserve (RPPN), part of the Embrapa Tabuleiros Costeiros Experimental Field in the same municipality (11°06′59.7″ S; 37°11′12.27″ W). Red propolis samples were obtained from the Union of Red Propolis Producers of the State of Alagoas (Uniprópolis), based in Maceió, Alagoas, Brazil. Following collection, fruit samples were dried in a ventilated oven at 45 °C for 48 h until reaching a final moisture content between 5% and 10%. All vegetable materials were then ground to a particle size between 16 and 32 mesh using Tyler series sieves. The processed samples were stored at −4 °C under a nitrogen atmosphere and protected from light until extraction.

### 4.3. Ultrasound-Assisted Pressurized Fluid Extraction (UAPFE)

Extractions were performed using a modified protocol based on Santos et al. (2018) [62]. A high-pressure extraction cell (20 mL) was loaded with a 3 g sample blend composed of equal proportions (1 g each) of *Hancornia speciosa* (mangaba), *Myrciaria floribunda* (cambuí), and red propolis. The extraction system was equipped with two high-pressure pumps to enable continuous solvent displacement and was connected to a horizontal ultrasonic bath (Ultronique, model Q5.9/40a) operating at a frequency of 40 kHz and a power output of 200 W, which provided both temperature control and sonication. Process variables were monitored using pressure transducers and universal indicators. Two extraction methodologies were employed: single-step and sequential. Single-step extractions were conducted at 50 bar and 60 °C using ethanol/water (80:20, *v*/*v*) and propane as individual solvents. Following a 5 min static stabilization period with ultrasound activation, the system maintained a continuous flow rate of 2 mL/min for 30 min per solvent. After each extraction, the system was depressurized via a needle valve, ultrasound was deactivated, and the extract was collected for analysis. Sequential extraction was designed to evaluate solvent selectivity. The sample blend was extracted in two stages, following solvent polarity: first with propane, then with ethanol/water (80:20, *v*/*v*). The system was pressurized to 50 bar and maintained at 60 °C with a flow rate of 2 mL/min. After a 5 min stabilization period (ultrasound on), the propane extraction was performed for 15 min. The system was then depressurized, and the procedure was repeated with ethanol/water under identical conditions for an additional 15 min, totaling 30 min of extraction time. Comparative analyses were conducted between the sequential and single-step extractions under equivalent experimental conditions (flow rate, pressure, temperature, solvent volume, and total extraction duration). Overall extraction yields (OEY) were calculated according to the following equation [68]:

Yield% = (DME/IM) × 100

DME (Dry Mass Extract). The solid material (g) was obtained after complete solvent removal from the liquid extract. This value represents the total amount of dry extractable compounds recovered.

IM (Initial Mass). The mass (g) of the starting sample was measured prior to any extraction procedure.

### 4.4. Characterization of UAPFE Extracts by Ultra-High-Resolution Mass Spectrometry (UHRMS)

The analysis of extracts obtained from both the sequential and single-step PLE + US processes was performed on an Exactive HCD Plus system (Thermo Scientific, Bremen, Germany), provided by CLQM. Samples were introduced by direct infusion using a 500 μL syringe (Thermo Scientific, Branchburg, NJ, USA). A heated electrospray ionization (HESI) source was used. For analysis, the extracts were dissolved in a mixture of ethanol and ultrapure water (1:20) at a concentration of 200 ppm. The system conditions are detailed in Table 3.

The results obtained from HESI(±)-FT Orbitrap MS analysis were processed using an advanced data interpretation workflow. Molecular formulas were assigned to detected ions using the Xcalibur Qual Browser software, version 3.1. For each *m*/*z* value, up to ten possible molecular formulas were considered, with a mass error threshold of less than 3 ppm. The elemental composition constraints applied were: ^13^C = 0–1; ^12^C = 5–100; ^1^H = 5–200; ^14^N = 0–5; and ^16^O = 0–20.

The list of ions was exported to Microsoft Excel, where the most probable molecular formula for each ion was selected using a proprietary algorithm developed by the Petroleum and Biomass Energy (PEB) Research Group. This algorithm incorporates analysis of the ^13^C isotopologue distribution and Kendrick mass defect (tolerance ± 0.001) to validate elemental composition assignments. Peaks associated with isotopic distributions were identified and excluded from further analysis [69]. As a quality control measure, only ions with a signal-to-noise ratio ≥ 3 were retained [70].

Given the complexity of the ultra-high-resolution mass spectrometry (UHRMS) dataset, comprising over 3000 detected ions—individual molecular analysis was impractical. To identify molecular patterns and differentiate extract samples obtained via UAPFE, Principal Component Analysis (PCA) was applied. PCA was performed using the PAST 4.0 software (freeware). Data were organized into matrices of neutral molecular formulas (5 × 1911), where rows represented extract samples and columns represented variables (relative intensity and molecular formula). Mean-centering was used as the preprocessing method.

### 4.5. Determination of Total Phenolic Compounds (TPC)

The TPC was determined following the protocol described by Costa et al. (2014) [71]. For the reaction system, 30 µL of each UAPFE extract was mixed with 150 µL of Folin–Ciocalteu (1:10) reagent and 120 µL of a sodium carbonate solution (7.5% *m*/*v*). The samples were then incubated in a water bath at 45 °C for 15 min. Absorbance was measured at 765 nm using a UV-Vis spectrophotometer. A blank solution containing all reagents except the extract was used for baseline correction. A visible color change from transparent to blue indicated the presence of phenolic compounds. Quantification was performed using a gallic acid calibration curve (10–100 mg/L). All determinations were conducted in triplicate, and results were expressed as µg of gallic acid equivalents (GAE) per mg of dry extract (DE).

### 4.6. Antioxidant Activity

#### 4.6.1. DPPH (2,2-Diphenyl-1-picrylhydrazyl) Assay

For the DPPH assay, the protocol of Costa et al., 2014 [71] was followed with some modifications. Briefly, 30 μL of UAPFE extract was mixed with 270 μL of a DPPH solution in ethanol. The decrease in DPPH was measured at 525 nm. DPPH scavenging activity was expressed in mg of trolox equivalents (TE)/μg of DE.

#### 4.6.2. Ferric Reducing Antioxidant Power (FRAP) Assay

The FRAP assay was performed according to Costa et al., 2014 [71] with minor modifications. In summary, 35 μL of vegetable blend extracts were mixed with 265 μL of FRAP solution (containing 0.3 M acetate buffer, 10 mM TPTZ solution, and 20 mM ferric chloride). After homogenization, the mixture was kept for 30 min at 37 °C protected from light interference. The absorbance was read at 595 nm. The calibration curve was prepared using ferrous sulfate (50–450 mg/L, r = 0.9998), and the ferric reducing antioxidant power was expressed in μg of ferrous sulfate equivalents (FSE)/mg of DE.

### 4.7. Cytotoxicity Assay (SRB Method)

Cytotoxicity was evaluated using the A549 human lung carcinoma cell line. Cells were seeded in 96-well microplates at a density of 1 × 10^4^ cells per well in 200 μL of culture medium and allowed to adhere for 24 h. After incubation, cells were treated with 100 μg/mL of each vegetable blend extract and maintained for 72 h under standard culture conditions. Following treatment, cells were fixed with 30% trichloroacetic acid (TCA) for 1 h at 4 °C. Plates were washed four times with distilled water and air-dried. Each well then received 100 μL of 0.057% (*w*/*v*) sulforhodamine B (SRB) solution prepared in 1% acetic acid, and the plate was incubated for 30 min at room temperature. Excess dye was removed by washing the wells four times with 1% acetic acid, followed by drying. To solubilize the bound SRB dye, 10 mM Tris(hydroxymethyl)aminomethane (TRIS) buffer (pH 10.5) was added to each well and incubated for 30 min. Absorbance was measured at 510 nm using a microplate reader (Synergy H1, BioTek, Winooski, VT, USA) to quantify cell viability [72].

### 4.8. Statistical Analysis

All experimental data are presented as mean ± standard deviation (SD). Statistical comparisons were performed using one-way analysis of variance (ANOVA) followed by Tukey’s post hoc test for multiple comparisons. Analyses were conducted using GraphPad Prism version 5.0 (GraphPad Software, San Diego, CA, USA). Differences were considered statistically significant at *p* < 0.05.

## 5. Conclusions

UAPFE proved to be an effective methodology for obtaining bioactive constituents from mangaba, cambuí, and red propolis blend. The use of solvents with varying polarities yielded extracts with distinct chemical composition and antioxidant activities, highlighting the superior selectivity of the ethanol/water (80:20) mixture. This solvent not only enhanced bioactivity but also aligns with the principles of sustainable development by being non-toxic and environmentally friendly. The UAPFE-SE and UAPFE-E extracts demonstrated significant antioxidant capacity and cytotoxic effects against lung carcinoma cells. These in vitro findings are relevant for exploring the therapeutic applications of these extracts. However, it is important to acknowledge a key limitation of the present study: the absence of cytotoxicity assays on non-cancerous human cell lines. To address this, future investigations will include dose–response analyses and testing on healthy cell lines, such as MRC-5, to determine IC_50_ values and calculate the therapeutic index. This will enable a more accurate assessment of selectivity and safety, which are critical parameters for validating the pharmacological potential of the extracts. Furthermore, a deeper understanding of the molecular and cellular mechanisms underlying the observed bioactivities is essential for advancing the development of these extracts as candidates for pharmaceutical, nutraceutical, or functional food applications. Such investigations will contribute to the rational design of multi-component formulations with enhanced efficacy and reduced off-target effects.

## 6. Patents

The work reported in this manuscript is the subject of a patent application filed with the National Institute of Industrial Property (INPI) under the number BR 10 2025 011628 6.

## Figures and Tables

**Table 1:** Representative compounds selected based on their statistical significance in PC1 loadings, highlighting the influence of solvent type and extraction process (sequential vs. one-step UAPFE) on the molecular composition of blend extracts.

Neutral Molecule	Detection Mode	UAPFE−SE (Area %)	UAPFE−SP (Area %)	UAPFE−E (Area %)	UAPFE−P (Area %)
C_12_H_18_O_12_	[M − H]^−^	3.611	0.051	1.951	0.007
C_12_H_22_O_9_	[M − H]^−^	2.384	ND	1.968	ND
C_15_H_12_O_5_	[M − H]^−^	2.136	0.083	2.251	ND
C_16_H_12_O_4_	[M − H]^−^	4.905	0.381	5.690	ND
C_16_H_12_O_5_	[M − H]^−^	4.271	ND	ND	ND
C_16_H_8_N_4_	[M − H]^−^	2.350	ND	ND	0.146
C_17_H_10_O_2_N_4_	[M − H]^−^	0.877	0.247	1.082	0.118
C_24_H_48_O_2_	[M − H]^−^	0.171	4.227	1.235	4.801
C_26_H_42_O_3_	[M − H]^−^	ND	2.820	ND	3.717
C_38_H_50_O_6_	[M − H]^−^	0.916	16.865	ND	20.930
C_6_H_12_O_6_	[M − H]^−^	2.824	0.072	1.345	0.022
C_6_H_8_O_7_	[M − H]^−^	8.399	0.253	5.940	ND
C_7_H_12_O_6_	[M − H]^−^	8.419	0.079	5.801	0.013
C_6_H_11_O_3_N_1_	[M − H]^−^	0.087	ND	0.064	ND
C_8_H_16_O_2_	[M − H]^−^	0.004	0.074	0.002	0.046

ND: not detected.

**Table 2:** Representative and Pharmacologically Relevant Metabolites extracted from the Blend via UAPFE Process.

Molecular Ion (*m*/*z*)	Molecular Formula	Cambui	Mangaba	Red Propolis	Classification	Biological Activity
271.0614 [M − H]^−^	C_15_H_12_O_5_	NI	Narigenin [16]	Narigenin [17]	Flavanone	Induces apoptosis and cell cycle arrest at G1 and G2/M phases; Inhibits metastasis via anti-angiogenic mechanisms and VEGF suppression; Reduces osteosarcoma growth [18]
271.0614 [M − H]^−^	C_15_H_12_O_5_	NI	-	Pinobanksin [17]	Flavanonol	Estrogenic activity in T47D cells via stimulation of proliferation [19]; Antioxidant, antiparasitic, and antibacterial properties [20].
271.0614 [M − H]^−^	C_15_H_12_O_5_	NI	-	3,4,2′,3′-Tetrahydroxychalcone [21]	Chalcona	Antioxidant activity [22].
267.0665 [M − H]^−^	C_16_H_12_O_4_	NI	NI	Formononetin [17,23]	Isoflavone	Modulates transcription factors and oncogenic pathways; reduces inflammation linked to cancer survival [24]; Induces apoptosis [24].
267.0665 [M − H]^−^	C_16_H_12_O_4_	NI	NI	Isoformononetin [24,25]	Isoflavone	Potentiates cytotoxicity in HeLa cells [19].
283.0613 [M − H]^−^	C_16_H_12_O_5_	NI	NI	2′-Hydroxyformononetin [17,26]	Isoflavone	Acts as an anti-inflammatory agent [26].
283.0613 [M − H]^−^	C_16_H_12_O_5_	NI	NI	Biochanin A [17,23,25]	Isoflavone	Inhibits the activity of invasive enzymes and induces cell cycle arrest and apoptosis [27].
283.0613 [M − H]^−^	C_16_H_12_O_5_	NI	NI	Thevetiaflavone [17,19]	Flavone	Exerts potential cytotoxic activity in various human cancer cell lines [28].
601.3534 [M − H]^−^	C_38_H_50_O_6_	NI	NI	Xanthochymol [29]	Benzophenone	Displays cytotoxic activity in a range of human tumor cell lines, such as A549 and vincristine-resistant KB cells [19]
601.3540 [M − H]^−^	C_38_H_50_O_6_	NI	NI	Gutiferone F [19,30]	Benzophenone	Induces apoptosis in certain tumor cells, such as hepatocellular carcinoma [19,31]
191.0197 [M − H]^−^	C_6_H_8_O_7_	Citric acid [9]	Citric acid [32]	NI	Carboxylic acid	Antioxidant agent [33].
191.0562 [M − H]^−^	C_7_H_12_O_6_	Quinic acid [9]	Quinic acid [32,34]	NI	Cyclitol	Functions as an antioxidant, antidiabetic, anticancer, antimicrobial, antiviral, anti-aging, protective, antinociceptive, and analgesic agent [35]
195.10185 [M + H]^+^	C_11_H_14_O_3_	N/F	NI	Methoxyeugenol [21,22]	Phenylpropanoid	Possesses anti-inflammatory properties and promising anticancer activity against endometrial cancer cells [36].

**Table 3:** Blend extract analysis parameters.

Parameter	HESI (−)	HESI (+)
Resolution	140 K @ *m*/*z* 200	140 K @ *m*/*z* 200
Capillary voltage	−3500 V	+4000 V
Chamber temperature	100 °C	100 °C
Capillary temperature	300 °C	300 °C
Sheath gas	15 a.u.	10 a.u.
Auxiliary gas	5 a.u.	10 a.u.
Counter flow gas	0 a.u.	0 a.u.
S-lens RF	40	40
Flow	25 µL min^−1^	25 µL min^−1^

## Data Availability

All original contributions from this study are detailed in the article. For further inquiries, please contact the corresponding author.

## Abbreviations

The following abbreviations are used in this manuscript:

CLQM	Multi-user Chemistry Laboratory Center
DE	Dry extract
DNA	Deoxyribonucleic acid
DPPH	2,2-diphenyl-1-picrylhydrazil
FRAP	Ferric Reducing Antioxidant Power
FT−Orbitrap MS	Fourier-Transform Orbitrap Mass Spectrometry
HCI	Hydrochloric acid
H−ESI	Heated-Electrospray ionization
*m*/*z*	Mass-to-load ratio
MAE	Microwave Assisted Extraction
ND	Not detected
NI	Unidentified
PCA	Principal Components Analysis
PLE	Pressurized liquid extraction
S/N	Signal/Noise
SFE	Supercritical Fluid Extraction
SRB	Sulforhodamine B
TPTZ	2,4,6-tris-(2-pyridyl)-s-triazine
TRIS	Tris(hydroxymethyl)aminomethane
UAE	ultrasound-assisted extraction
UAPFE	Pressurized liquid fluid extraction assisted ultrasound
UAPFE−E	Ultrasound-assisted pressurized liquid extraction with ethanol/water
UAPFE−P	Ultrasound-assisted extraction with pressurized liquid using propane
UAPFE−SE	Ultrasound-assisted pressurized liquid extraction with sequential ethanol/water
UAPFE−SP	Ultrasound-assisted pressurized liquid extraction with sequential propane
UHRMS	Ultra-High Resolution Mass Spectrometry
UV-Vis	Ultraviolet-Visible
